# Xenotransplantation of pediatric low grade gliomas confirms the enrichment of *BRAF* V600E mutation and preservation of *CDKN2A* deletion in a novel orthotopic xenograft mouse model of progressive pleomorphic xanthoastrocytoma

**DOI:** 10.18632/oncotarget.20713

**Published:** 2017-09-08

**Authors:** Mari Kogiso, Lin Qi, Holly Lindsay, Yulun Huang, Xiumei Zhao, Zhigang Liu, Frank K. Braun, Yuchen Du, Huiyuan Zhang, Goeun Bae, Sibo Zhao, Sarah G. Injac, Mary Sobieski, David Brunell, Vidya Mehta, Diep Tran, Jeffrey Murray, Patricia A. Baxter, Xiao-Jun Yuan, Jack M. Su, Adekunle Adesina, Laszlo Perlaky, Murali Chintagumpala, D. Williams Parsons, Ching C. Lau, Clifford C. Stephan, Xinyan Lu, Xiao-Nan Li

**Affiliations:** ^1^ Department of Pediatrics, Baylor College of Medicine, Texas Children's Cancer Center, Texas Children's Hospital, Houston, TX, USA; ^2^ Department of Neurosurgery, The First Affiliated Hospital, Soochow University, Suzhou, China; ^3^ Department of Ophthalmology, First Affiliated Hospital of Harbin, Medical University, Harbin, China; ^4^ Department of Radiotherapy, Hunan Cancer Hospital, The Affiliated Cancer Hospital of Xiangya School of Medicine, Central South University, Changsha, China; ^5^ Center for Translational Cancer Research, Institute of Biosciences and Technology, Texas A&M College of Medicine, Houston, TX, USA; ^6^ Department of Pathology, Baylor College of Medicine, Texas Children's Hospital, Houston, TX, USA; ^7^ Department of Hematology and Oncology, Cook Children's Medical Center, Fort Worth, TX, USA; ^8^ Department of Hematology and Oncology, Xinhua Children's Hospital, Shanghai, China; ^9^ Department of Pathology, Northwestern University Feinberg School of Medicine, Chicago, IL, USA

**Keywords:** low grade glioma, orthotopic xenograft, cancer stem cell, *BRAF* V600E, CDKN2A

## Abstract

To identify cellular and molecular changes that driver pediatric low grade glioma (PLGG) progression, we analyzed putative cancer stem cells (CSCs) and evaluated key biological changes in a novel and progressive patient-derived orthotopic xenograft (PDOX) mouse model. Flow cytometric analysis of 22 PLGGs detected CD133^+^ (<1.5%) and CD15^+^ (20.7 ± 28.9%) cells, and direct intra-cranial implantation of 25 PLGGs led to the development of 1 PDOX model from a grade II pleomorphic xanthoastrocytoma (PXA). While CSC levels did not correlate with patient tumor progression, neurosphere formation and *in vivo* tumorigenicity, the PDOX model, IC-3635PXA, reproduced key histological features of the original tumor. Similar to the patient tumor that progressed and recurred, IC-3635PXA also progressed during serial *in vivo* subtransplantations (4 passages), exhibiting increased tumor take rate, elevated proliferation, loss of mature glial marker (GFAP), accumulation of GFAP^−^/Vimentin^+^ cells, enhanced local invasion, distant perivascular migration, and prominent reactive gliosis in normal mouse brains. Molecularly, xenograft cells with homozygous deletion of *CDKN2A* shifted from disomy chromosome 9 to trisomy chromosome 9; and *BRAF* V600E mutation allele frequency increased (from 28% in patient tumor to 67% in passage III xenografts). *In vitro* drug screening identified 2/7 *BRAF* V600E inhibitors and 2/9 *BRAF* inhibitors that suppressed cell proliferation. In summary, we showed that PLGG tumorigenicity was low despite the presence of putative CSCs, and our data supported GFAP^−^/Vimentin^+^ cells, *CDKN2A* homozygous deletion in trisomy chromosome 9 cells, and *BRAF V600E* mutation as candidate drivers of tumor progression in the PXA xenografts.

## INTRODUCTION

Pediatric low grade gliomas (PLGGs) are slow growing tumors accounting for 1/3 of all childhood brain tumors [1]. Although complete surgical removal results in cure in >90% of patients, some tumors still recur [1–3], especially after sub-total resection. Currently, driver(s) of recurrence and malignant progression remain to be elucidated. Mouse models that replicate key biological features of PLGG are highly desired to identify mechanism of recurrence/malignant degeneration and enable pre-clinical studies of PLGG. We have shown that direct injection of fresh surgical specimens into anatomically-matched locations in the brains of immunodeficient mice facilitates establishment of clinically-relevant orthotopic xenograft mouse models that replicate the histology, invasive growth, and key genetic features of primary patient tumors [4–8]. The added advantage of patient-derived orthotopic xenograft (PDOX) mouse model is that the normal brain responses toward xenograft growth, which is difficult to obtain from patient surgical samples, can be analyzed simultaneously together with brain tumor cells. PDOX mouse models of PLGGs, however, have not been previously established.

Accumulating evidence demonstrates that cancer stem cells (CSCs) play an important role in tumorigenicity, cancer initiation and recurrence [9–14]. CD133 and CD15 are two well-characterized cell surface markers that define pediatric glioblastoma and medulloblastoma CSCs [8, 9, 14–18]. Despite ongoing controversies about the relative abundance and specificity of these markers [19–21], CD133^+^ brain tumor stem cells are chemotherapy- and radiation-resistant [13, 22], and their frequency correlates with adverse survival in adult glioma [23]. In contrast, little is known about CSCs in low grade tumors. Only a few cases have been analyzed for CD133^+^ cells, revealing variable abundance ranging from undetectable [24] to 37% [25]. The content and function of CD15^+^ cells in PLGGs is still unknown.

Genetic analysis identified *BRAF* as a frequent mutation target in PLGGs, including *BRAF* V600E mutation [26–32], duplication [33] and gene fusion [28–34]. *BRAF* V600E mutation were found in WHO grade II PXA (66%), PXA with anaplasia (65%), grade I GG (18%) and grade I PA (9%) [29]. Homozygous deletions involving the CDKN2A/p14ARF/CDKN2B loci were detected in 60% of PXA [35] and 71% of malignant astrocytomas [36]. These reports suggest contribution of *BRAF* V600E mutation and *CDKN2A* deletion to tumor progression and should be targeted. Indeed, multiple novel inhibitors against *BRAF* V600E mutation have been developed and entered into clinical trials in patients with advanced melanoma, hairy cell leukemia, and thyroid cancers [37–40]. Developing new PLGG models replicating such druggable mutation would be highly desired not only to understand the functional role of *BRAF* V600E mutation in driving PLGG recurrence, but also for future examination of drug resistance as has been noted in melanomas [37].

In this report, our goals were to determine if PDOX models can be established from low grade gliomas, whether CSCs are present in PLGG and if their frequencies correlate with *in vitro* self-renewal, *in vivo* formation of orthotopic xenografts, and clinical tumor recurrence. To gain insight into *in vivo* tumor evolution and progression, we examined if the histopathological features and, more importantly, the progression nature of the original patient tumor were replicated in the PDOX tumors during long-term serial subtransplantations in mouse brains, followed by the analysis of the underlying cellular and molecular (e.g. *BRAF V600E* mutation and *CDKN2A* deletion) changes in tumor cells and in the host normal brain cells that drove or accompanied the PDOX tumor progression to identify new therapeutic targets.

## RESULTS

### The overall yields of tumor cells from childhood LGG were low

Despite extensive collaborative effort, the tumor tissues obtained for PLGGs were still limited, frequently less than 3 × 3 × 3 mm^3^ (Table 1). Using a combination of mechanical dissociation and combined collagenase/halogenase enzymatic digestion, we were able to collect viable tumor cells up to 4.3 × 10^6^ cells (1.3 × 10^6^ ± 1.1 × 10^6^). The number of assays per PLGG sample was therefore performed depending on the tumor cell availability.

**Table 1:** Summary of clinical information, tumor cell yield and intra-cranial tumor formation of PLGG tumors

No.	Tumor ID	Age, Gender	Dx, WHO Grade	Tumor cell number(x 10^6^)		Site of injection	Tumor formation in mouse brains	
				Total	Injected/mouse		Total	With Tumor
1	ST-1267^¶^	1 y, M	PA, I	1	0.1	ICb	5	0
2	ST-1342^¶^	3 y, F	PA, I	1.8	0.1	ICb	2	0
3	ST-1610^¶^	15 y, M	PA, I	1	0.1	ICb	5	0
4	ST-1823	11 y, M	PA, I	0.38	0.1	ICb	3	0
5	ST-1828	8 y, M	PA, I	0.33	0.1	ICb	3	0
6	ST-2076	8 y, M	PA, I	1.05	0.1	ICb	5	0
7	ST-2109^*^	4 y, F	PA, I	2.68	0.16	ICb	10	0
8	ST-2354^*¶^	6 y, M	PA, I	3.6	0.25	IC	8	0
9	ST-2431^*^	4 y, F	PA, I	0.72	0.1	IC	5	0
10	ST-2446^*¶^	1 y, M	PA, I	1.76	0.085	ICb	3	0
11	ST-2794^*¶^	5 y, F	PA, I	1.12	0.16	ICb	3	0
12	ST-2879^*¶^	12y, M	PA, I	1.1	0.1	ICb	5	0
13	ST-2990^¶^	11 y, F	PA, I	1.54	0.2	ICb	5	0
14	ST-3016	9 y, M	PA, I	0.45	0.12	ICb	3	0
15	ST-3278^*^	10 y, F	PA, I	2	0.15	ICb	5	0
16	ST-3526^*¶^	9 y, M	PA, I	4.3	0.15	ICb	10	0
17	ST-3580^¶^	13 y, F	PA, I	1.12	0.2	ICb	5	0
18	ST-3593	5 y, M	PA, I	1.55	0.15	ICb	10	0
19	ST-3648^¶^	6 y, M	PA, I	2.7	0.28	IC	7	0
20	ST-4109^¶^	4 y, F	PA, I	2	0.2	ICb	5	0
21	ST-4666^¶^	1 y, F	PA, I	1.85	0.15	ICb	10	0
22	ST-1756^*¶^	9 y, M	PA, I	0.025	No			
23	ST-2270^*^	9 y, M	PA, I	0.1	No			
24	ST-2439^*^	8 y, M	PA, I	0.058	No			
25	ST-2728^¶^	17 y, M	PA, I	0.03	No			
26	ST-3490	13 y, M	PA, I	0.01	No			
27	ST-3510^¶^	10 y, F	PA, I	0.05	No			
28	ST-5293^¶^	8 y, F	PA, I	0.05	No			
29	ST-5336^*¶^	5 y, M	PA, I	0.4	No			
30	ST-4461	11 y, F	AST, II	0.31	0.1	ICb	3	0
31	ST-4388^*¶^	11 y, F	AST, II	0.03	No			
32	ST-4034^¶^	6 y, F	GG	0.06	No			
33	ST-2829^*¶^	12 y, M	GG	0.37	0.063	ICb	5	0
34	ST-4524	13 y, F	GG	2.55	0.25	IC	10	0
35	ST-4710^¶^	13 y, F	GG	0.36	No			
36	ST-3635^*^	10 y, F	PXA, II	3.48	0.1	IC	7	2

**Note:** ^*^Tested for neurosphere formation, ^¶^Analyzed with FCM for CSC markers, **Dx**= diagnosis, **ICb**=Intra-cerebellar, **IC**=Intra-cerebral, **PA**=Pilocytic astrocytoma, **AST**=astrocytoma, **GG**=Ganglioglioma, **PXA** = Pleomorphic Xanthoastrocytoma

### Attempts to establish neurosphere and monolayer cultures from patient tumors

To examine if PLGGs contain cells able to form neurospheres *in vitro*, dissociated cells from 15 tumors (Table 1) were plated in serum-free medium containing Neurobasal medium, EGF/bFGF to favor CSC growth [7, 8, 15]. Sustained neurosphere growth was unsuccessful from any tumor after 19 to 57 (37.6 ± 15.2) days observation. Additionally, 11 patient tumors with >2 × 10^6^ cells were incubated in FBS-based media. Again, none attached or expanded in culture. These data suggested that PLGG cells harvested directly from patients did not readily adapt to *in vitro* growth conditions and failed to proliferate.

### Only 1 of 25 PLGGs formed orthotopic xenograft tumors

**Figure 1:**
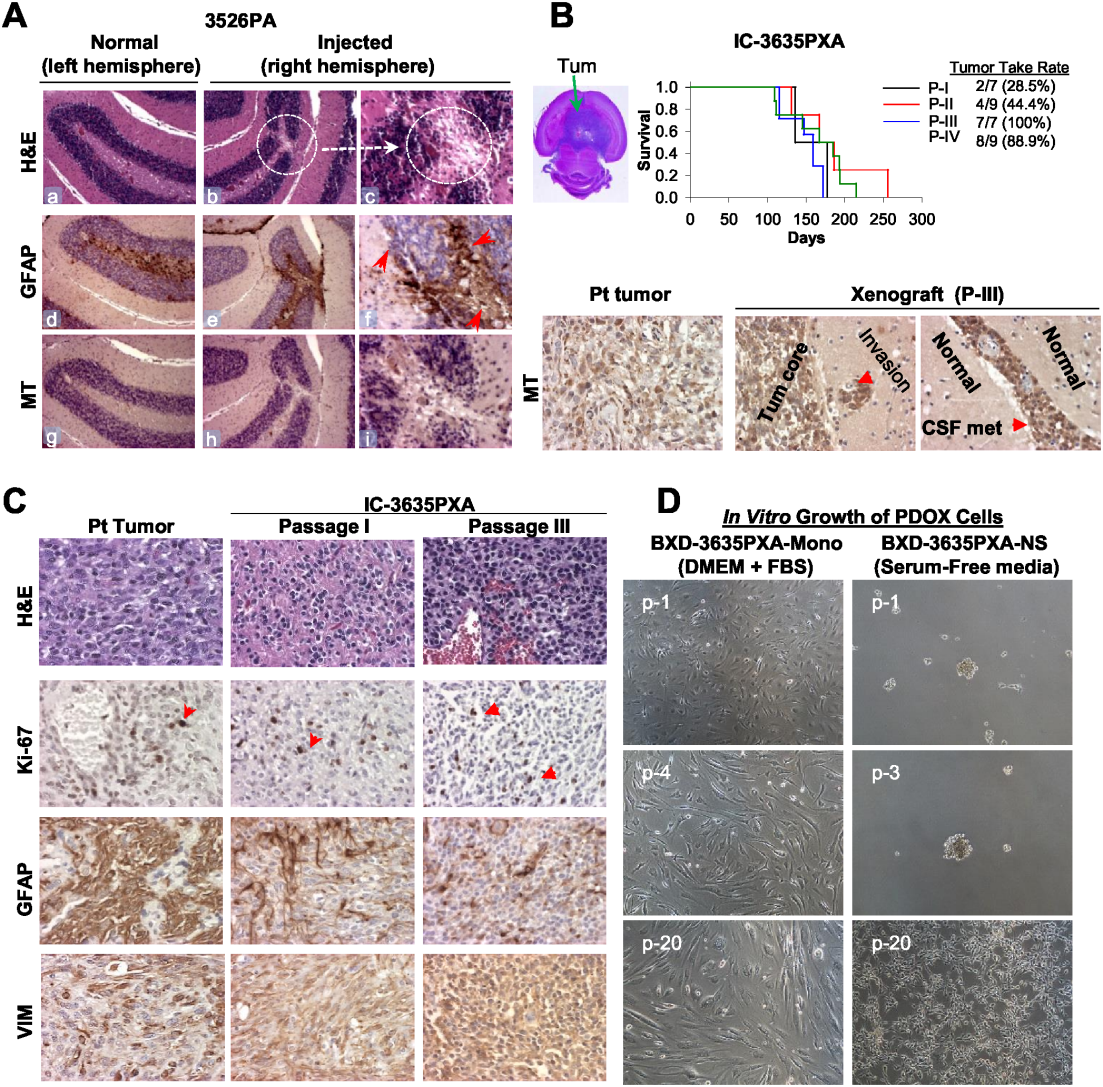
Establishment of *in vivo* and *in vitro* models of PLGGs **(A)** H&E and IHC staining of mouse brain show scars of surgical implantation without tumor formation, disturbed granular layer neurons (*b-c, circle*), and reactive mouse astrocytes (monoclonal antibodies against GFAP) (*e-f*). Human tumor cells detected with human-specific antibodies against mitochondria (MT) (*h-i*). Magnification (x10: *a, b, d, e, g, h* and x40: *c, f, i*). **(B)** H&E stained cross section of IC-3635PXA (*top left image*). Log-rank analysis of animal survival times during serial sub-transplantation from passage I (P-I) to IV (P-IV) (*top right panel*). IHC of tumor cells with human-specific MT antibodies (*lower panel*). **(C)** Histopathological features of IC-3635PXA xenograft tumors (at passage I and III) compared with patient tumor (magnification: x20). **(D)** Morphology of cultured PDOX cells in FBS-based media (BXD-3635PXA-mono) and serum-free media supplemented with EGF/bFGF (BXD-3635PXA-NS) from passages 1 (p-1) to 20 (p-20) (magnification: x10).

We tested if rapid return of PLGG cells to anatomically-matched locations in mouse brains would form tumor. 20 cerebellar tumors and 5 cerebral PLGGs were implanted into matching locations in mouse brains either intra-cerebellarly (ICb) or intra-cerebrally (IC) (Table 1) as we described previously. No visible tumor formation was detected in 24 of 25 PLGGs after average of 230 days observation. H&E staining of paraffin-embedded brains also did not detect tumors, although scarring and disturbed normal brain structure indicative of previous surgical implantation were seen (Figure 1A). Subsequent IHC examination revealed reactive astrocytes with strong GFAP positivity surrounding the needle track but did not detect human cells in mouse brains using human-specific MT antibodies, further confirming the lack of xenograft tumor formation (Figure 1A). Only IC implantation of 3635 pleomorphic xanthoastrocytoma (PXA) patient tumor formed orthotopic xenograft tumors; this model was designated IC-3635PXA and was serially sub-transplanted in mouse brains four times (passage IV) (Figure 1B). Similar to patient tumor, xenograft cells from passages I to III were positive for human-specific MT in tumor core, invasive foci, and disseminated cells (Figure 1B), validating the human origin of xenograft tumors.

The PDOX model-initiating patient tumor was pathologically assessed at diagnosis by the local anatomic pathologist and was subsequently reviewed by our institutional neuro-pathologist. The tumor was confirmed to be a pleomorphic xanthoastrocytoma (2016 Central Nervous System (CNS) World Health Organization (WHO) grade II) as it was moderately cellular and infiltrative but with a low proliferation index and no necrosis or anaplasia [41]. Rare sections of the tumor were suspicious for developing vascular proliferation. Pathologic examination of xenograft passage I revealed the presence of a 2016 CNS WHO grade II PXA with no vascular proliferation or necrosis and very low mitotic activity [41]. Xenograft passage II tumors remained grade II but demonstrated increased cellularity, though the proliferation index remained low. At xenograft passage III, tumors had a notable pathologic shift with developing of higher grade features including regional necrosis pseudopalisading necrosis, increased vascular and endothelial proliferation, increased tumor cell infiltration into normal brain. The tumor's proliferative index had increased from <5% to approximately 20%. Xenograft passage IV had even further increases in tumor cellularity and had unequivocally transformed into a high grade tumor.

### Tumor take rate of IC-3635PXA increased during serial *in vivo* sub-transplantations

Initial tumor take rate upon implantation of patient tumor into SCID mice was low, as only 2 of 7 (28.5%) mice initially implanted with 3635PXA patient tumor formed tumors. Over *in vivo* sub-transplantations, tumor take rate steadily increased: 28.5% (2/7) in passage I, 44.4% (4/9) in passage II, 100% (7/7) in passage III, and 88.9% (8/9) in passage IV (Figure 1B). Presence of normal cells in the patient tumor and the multiple round selection of “pure” and more aggressive clonal cell populations might have played a role.

### PDOX tumor cells survived and proliferated *in vitro*

We also attempted to establish cell lines from IC-3635PXA xenografts. Monolayer cells, labeled Baylor xenograft derived (BXD)-3635PXA-mono, grew in FBS-based media, although the proliferation rate of these cells was slow (21-28 days/passage and reached passage 25 after 586 days). In serum-free media, 3635PXA cells initially formed neurospheres (BXD-3635PXA-NS) but after passage 5 became attached, exhibiting a star-like morphology with occasional formation of small clusters (7-18 days/passage and reached passage 32 after 586 days) (Figure 1D). PDOX tumor cells may have a better chance of survival *in vitro* than the patient tumors.

### PLGGs contain high levels of CD15^+^ cells and low levels of CD133^+^ cells

**Figure 2:**
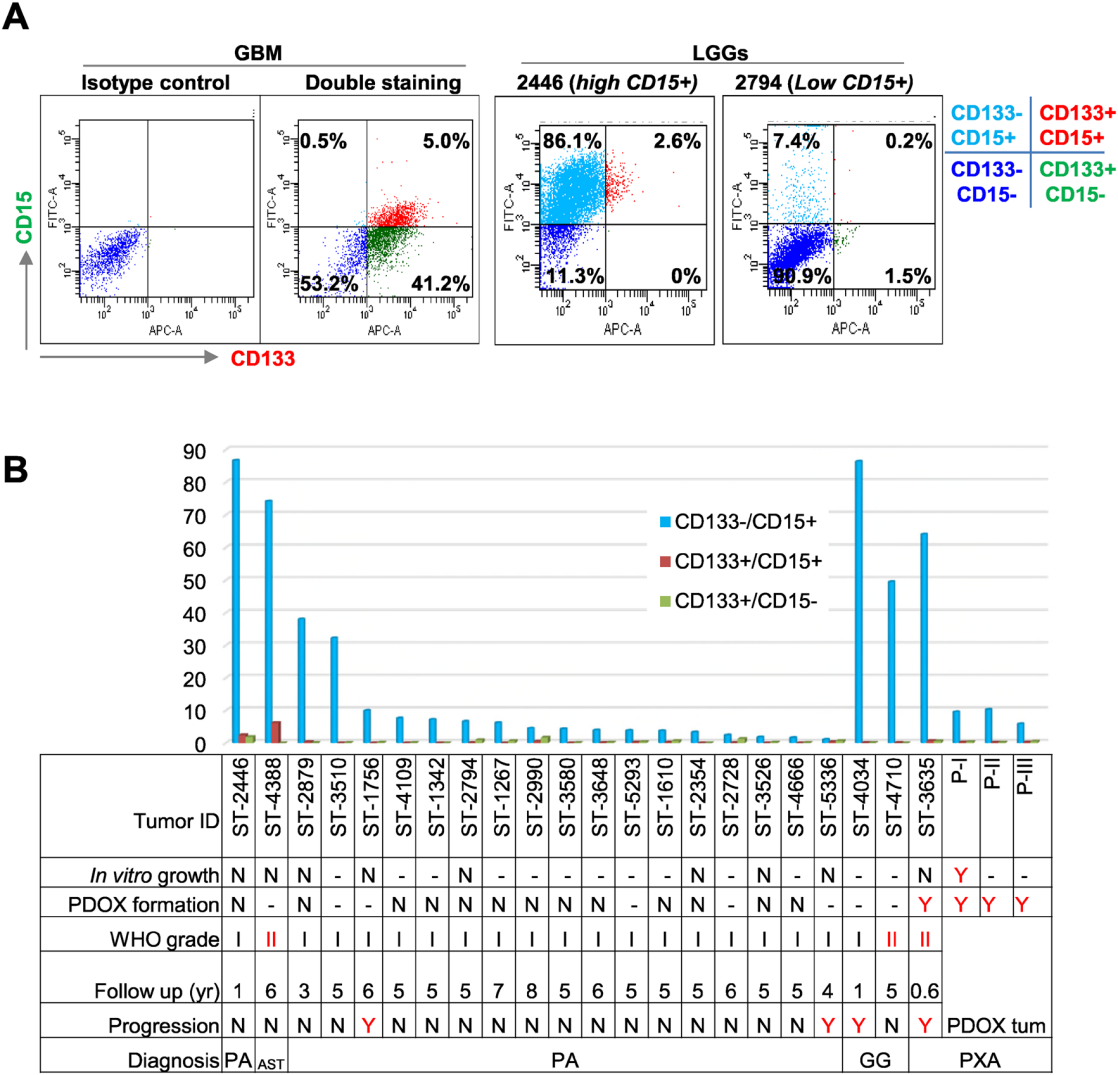
Analysis of CD133^+^ and CD15^+^ cells **(A)** Representative graph showing successful double staining of CD133 and CD15 in GBM (*left 2 panels*) and PLGGs with high (*middle panel*) and low (*right panel*) CD15^+^ cells. **(B)** Summary graph showing relative abundance of mono- and dual-positive (CD133 and CD15) cells related to tumor cell growth, PDOX formation, pathological grade, years of follow up, and status of progression. Due to limited viable tumor cell yields, not all samples were tested with all assays. “-”: not tested; N = did not grow/no progression, Y = grew in culture or *in vivo* or progressed.

To determine if lack of putative CSCs was the cause of low xenograft tumor formation, we assessed CD133 and CD15, two common brain tumor CSC markers, in 22 patient tumors using flow cytometry (FCM) (Figure 2). PLGGs were found to have abundant CD133^−^/CD15^+^ cells (20.7 ± 28.9%) (Figure 2A and 2B). In 3 of 18 (17%) pilocytic astrocytomas (PAs), 1 of 1 (100%) grade II astrocytoma (AST), 2 of 2 (100%) gangliogliomas (GGs), and 1 of 1 (100%) PXA, CD133^−^/CD15^+^ cells accounted for >30% of the total cell population (Figure 2B), demonstrating CD15^+^ cells in PLGGs for the first time. Compared with high levels of CD133^+^/CD15^−^ cells in a childhood GBM xenograft tumor included as positive control (Figure 2A), only low levels (0.46 ± 0.57%) of CD133^+^/CD15^−^ PLGG cells were detected in 22 tumors (Figure 2B).

We subsequently analyzed CD133^+^ and/or CD15^+^ levels in 3635PXA and the resulting xenograft tumors (passages I through III). Similar to other PLGGs, CD133^+^/CD15^+^ and CD133^+^/CD15^−^ were barely detectable (<1%), while CD133^−^/CD15^+^ cells were the major subpopulation, accounting for 64.1% in patient tumor (Figure 2B). CD133^−^/CD15^+^ xenograft tumor cells decreased to 9.6% in passage I, 10.3% in passage II and 5.9% in passage III (Figure 2B), suggesting that they may not be driving *in vivo* tumor formation.

In order to correlate the content of putative CSC cells with clinical tumor progression, clinical outcomes of 22 patients with PLGG were followed for up to 6 (4.8±1.6) years. Among the four progressive tumors, one GG and one PXA had high CD15^+^ cells (>60%) but two PAs harbored only 10% and 1% CD15^+^ cells (Figure 2B). Altogether, these data suggested that tumorigenicity in mouse brains and clinical progression of PLGGs may not be solely driven by putative CSCs.

### BRAF V600E mutation increased during IC-3635PXA progression

**Figure 3:**
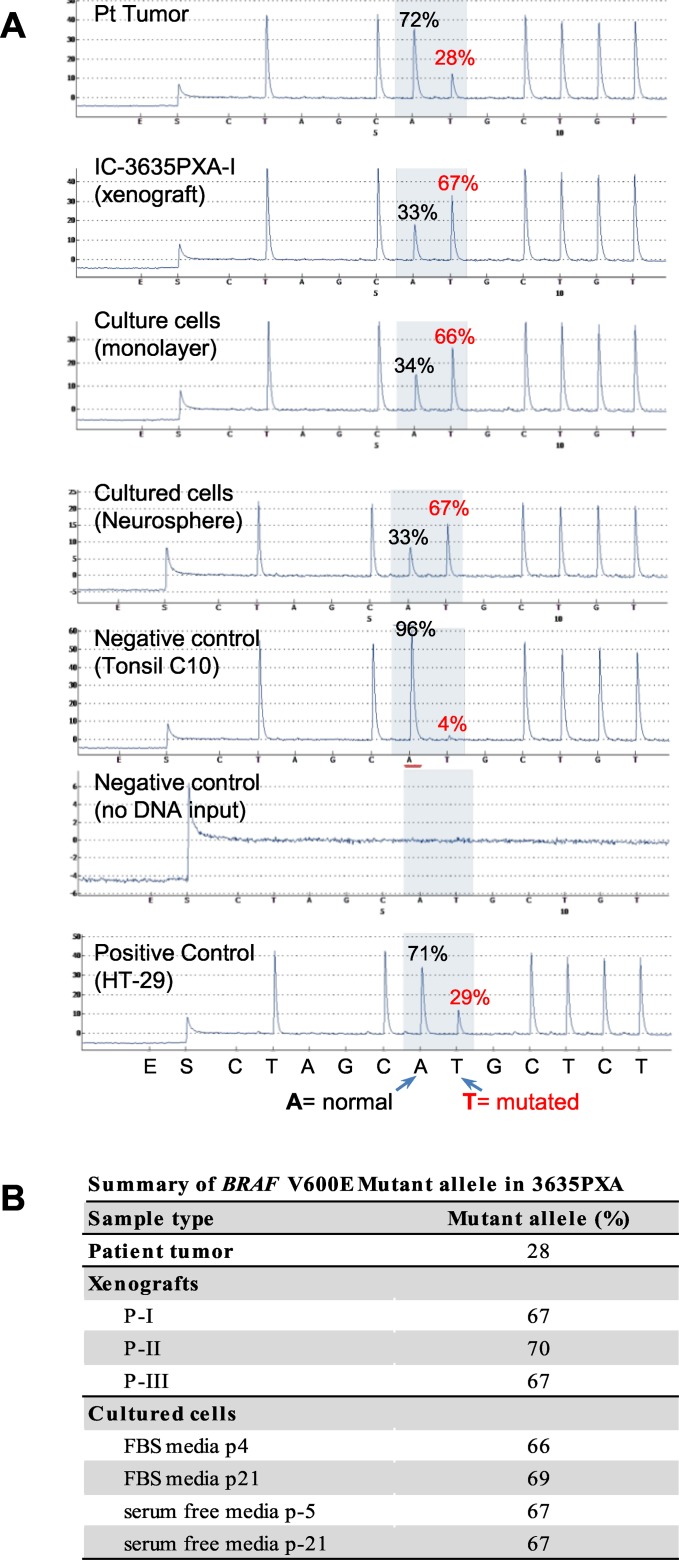
Quantitative analysis of *BRAF* V600E mutation allele frequency using pyrosequencing **(A)** Representative pyrograms indicating A (wild type) to T (mutated) substitution in percent for 3635PXA patient tumor, xenograft passage I as well as *in vitro* cultured 3635PXA cells (monolayer and neurospheres) are shown. Pyrosquencing was control by including a positive control (HT-29) as well as negative controls with either no mutation (Tonsil) or no DNA. Percent (%) A/T is calculated using pyromark analysis software. **(B)** Allele frequency of *BRAF* V600E mutation in patient tumor, xenografts and cultures cells of 3635PXA.

Genomic DNA from 3635PXA patient, xenograft tumors and cultured cells was subjected to quantitative mutation detection using *BRAF* V600E specific pyrosequencing. Compared to 28% in patient tumor, *BRAF* V600E mutant allele frequency increased in xenografts (67%, 70%, and 67% in passage I, II and III, respectively) (Figure 3A and 3B). *In vitro* xenograft tumor cell cultures also had high *BRAF* mutation frequencies with 66% (p-4), 69% (p-21) in monolayer and 67% (p-5 and p-21) in neurospheres. These observations suggest that *BRAF* V600E mutation plays an important role in tumor progression, although the molecular mechanisms underlying the increased allele frequency of *BRAF* mutation frequency remains to be determined.

### IC-3635PXA PDOX showed persistent CDKN2A deletion and increasing trisomy 9

**Figure 4:**
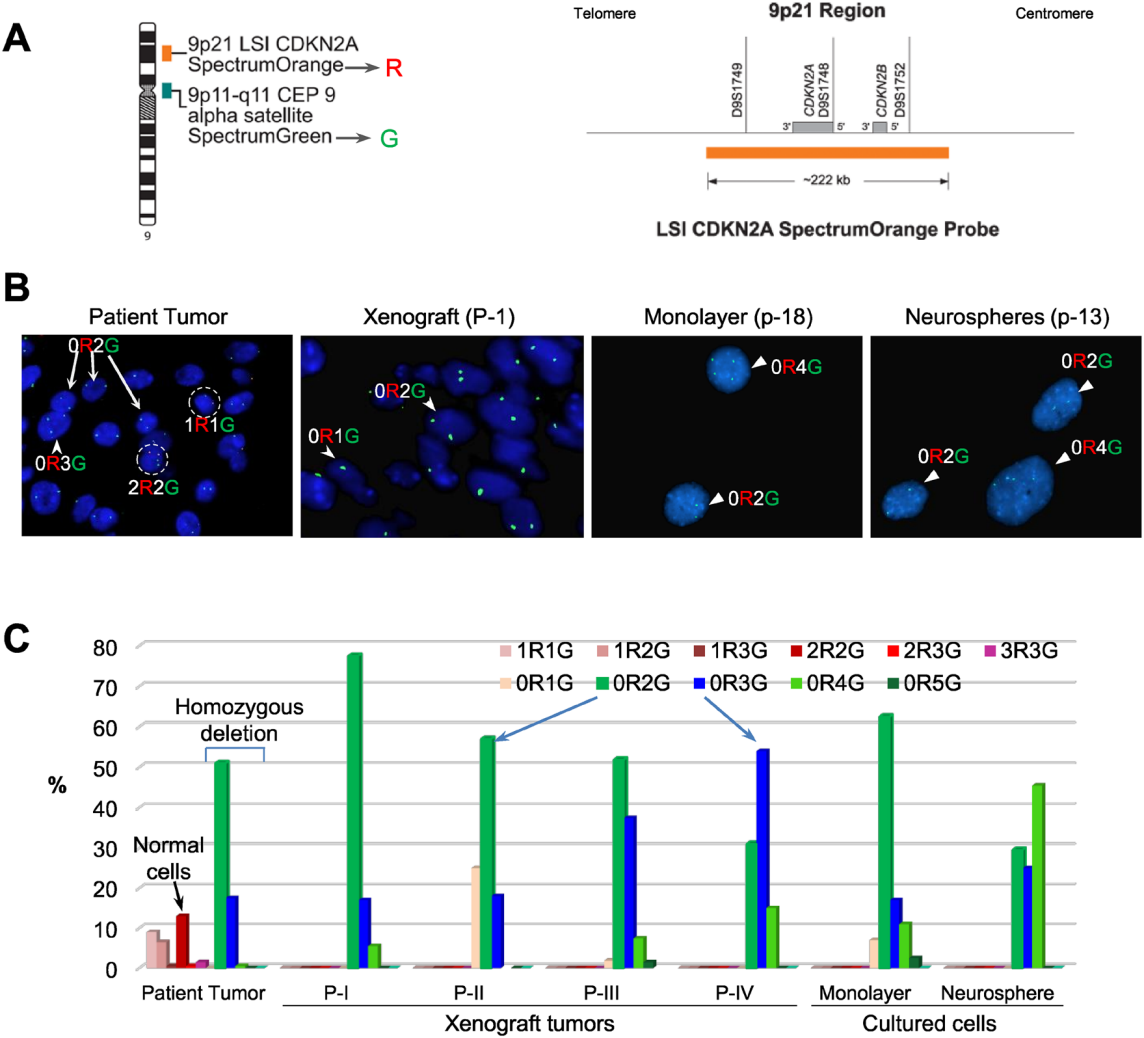
FISH validation of *CDKN2A* deletion **(A)** Location and the coverage of *CDKN2A* by spectral orange (*R* = red) on chromosome 9. The alpha satellite on the centromere of chromosome 9 is labeled with SpectrumGreen (*G* = green) and used as the reference control. **(B)** Images of FISH hybridization in paraffin sections (patient tumor and xenograft) and in cultured cells (monolayer and neurospheres). Normal cells with two copies of chromosome 9 centromere (green) and two copies of *CDKN2A* (red) highlighted in circle and labelled as 2R2G. Loss of *CDKN2A* (no red = 0R) were found in disomy (0R2G), trisomy (0R3G) and quadrosomy (0R4G) tumor cells (*arrow*s). **(C)** Graph showing the relative percentage of cells with or without *CDKN2A* deletion. For each sample, 200 cells were counted under high magnification (10 × 60). Compared with the patient tumor, in which *CDKN2A* were still present in a small fraction of cells (mostly 2R2G), xenograft tumors and both the cultured cells were enriched with homozygous deletion of *CDKN2A* (0R1G to 0R5G). Note the gradual decrease of disomy chromosome 9 (0R2G) and increase of trisomy 9 (0R3G) (*arrows*) over *in vivo* sub-transplantations of IC-3635PXA.

Oncogenic *BRAF* mutation and *CDKN2A* inactivation characterize a subset of pediatric malignant gliomas [36]. We thus examined if *CDKN2A* deletion played a role in IC-3635PXA progression. Utilizing Vysis LSI *CDKN2A* SpectrumOrange/CEP 9 SpectrumGreen Probes (Figure 4A), we analyzed *CDKN2A* status in paraffin sections of patient and xenograft tumors and cultured cells derived from IC-3635PXA. In the patient sample, 13% of the cells contained 2 red (R=*CDKN2A*) and 2 green (G=chromosome 9 centromere) (2R2G in Figure 4B) that are found in normal cells, most patient tumor cells (87%) exhibited homozygous deletion either with disomy chromosome 9 (0R2G) (51%) or trisomy 9 (0R3G) (17.5%). As expected, there were no normal 2R2G human cells in xenograft tumors. In xenograft passage I, homozygous deletion of *CDKN2A* was found in 77.5% cells with 0R2G, 17% cells with 0R3G, and 5% cells with quadrisomy 9 (0R4G). Subsequent analysis of xenograft tumors revealed a gradual decrease of 0R2G cells with *CDKN2A* deletion, i.e. 58%, 51%, and 31% in passages II, III and IV, accompanied by increased 0R3G cells from 18% in passage II, to 37% in passage III and 54% in passage IV (Figure 4C). These data suggested that a sub-population of trisomy chromosome 9 PLGG cells with *CDKN2A* deletion gained growth advantage over disomy 9 cells.

Cultured xenograft tumor cells also maintained the homozygous *CDKN2A* deletion (Figure 4B). The relative abundance of 0R2G decreased from 62.5% in the monolayer cells to 29% in neurospheres while 0R3G and 0R4G cells increased from 17% to 25%, and from 11% to 45%, respectively (Figure 4C). These data demonstrated that homozygous deletion of *CDKN2A*, found in 68.5% of 3635PXA patient tumor cells, was well maintained and increased to 100% in the PDOX model and cultured xenograft cells; interestingly, monolayer cells were enriched with disomy 9 cells while neurospheres favored the growth of trisomy and quadrisomy 9 tumor cells.

### PDOX tumor evolution was paralleled by loss of GFAP and gain of Vimentin (VIM) expression

**Figure 5:**
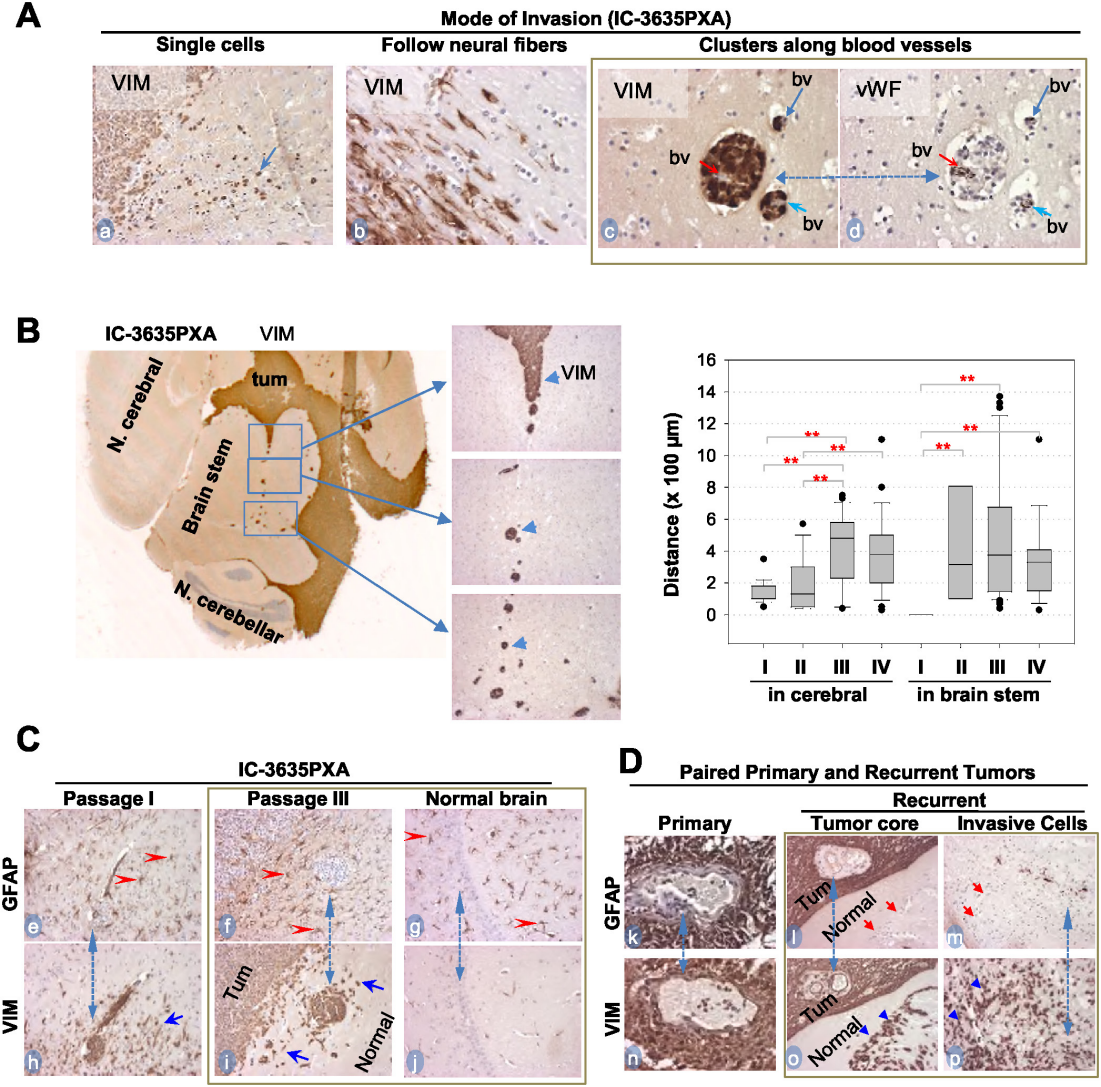
*In vivo* tumor invasion and host responses detected with IHC **(A)** Modes of IC-3635PXA invasion in mouse brains. Tumor cells positively stained with Vimentin (VIM) (*arrows*, *a-c*), and blood vessels (bv) with vWF (*d*). Same area in two consecutive sections was included (*c* and *d*, *dotted line with dual arrow heads*). **(B)** Images showing long-range perivascular invasion (*left panel*) and quantitative analysis perivascular migration (*right panel*) (^**^ *P*< 0.01). Tumor cells positively stained with VIM (*arrow heads*). **(C)** IHC showing mutually exclusive positivity between GFAP (marker for mature glial cells) (*red arrowheads, e-g*) and VIM in tumor mass (Tum) and in invasive satellites and single cells (*blue arrowheads*, *h* and *i*). Note presence of reactive astrocytes in normal brain tissues (*g*) without presence of tumor cells (*j*). Matched areas in consecutive sections were used (*dotted line with dual arrow heads)* for GFAP and VIM staining (magnification x 20). **(D)** IHC of paired primary and recurrent PA confirming invasive cells to be GFAP- (*red arrow*, *l* and *m*) and VIM^+^ (*blue arrowheads*, *o* and *p*) Magnification (x40: *k* and *n*; x20: *l, m, o, p*).

To identify cellular changes accompanying increased tumor take rate and the increased *BRAF* mutation, we performed IHC staining of PDOX tumors (Figure 1C and Table 2). First, compared with low cell proliferation index (Ki-67) of <2% in patient tumor, xenograft tumors from IC-3635PXA exhibited Ki-67 positivity at 5%-10% in passage I and 5%-15% in passage III. Second, while the majority of patient tumor cells (>95%) were strongly positive (+++) for GFAP, a marker of mature/differentiated glial cells, there was a steady decrease of GFAP^+^ cells in PDOX tumors (26-50%). Third, expression levels of VIM, a marker of intermediate filament associated with poor prognosis and tumor invasion [42–44], increased. While high (+++) VIM expression was seen in only a low percentage (~10-30%) of patient tumor cells, nearly all xenograft cells exhibited strong (+++) positivity in passages I and III. In addition to cells in the tumor core, invasive tumor cells (single cells, micro-satellites, and perivascular spread) were also strongly (++++) VIM positive (Figure 5A). These data suggested that a sub-population of GFAP^−^/VIM^+^ cells were enriched during PDOX tumor formation, sub-transplantation, and tumor progression *in vivo*.

**Table 2:** Summary of immunohistochemical characteristics of 3635PXA

Target Phenotypes	Marker	Patient Tumor	Xenograft(passage I)		Xenograft(passage III)	
			TC	INV	TC	INV
**Proliferation**	**Ki-67**	++(1-5%)	++(5-10%)	++(1-10%)	++(5-15%)	++(5-15%)
**Astrocyte**	**GFAP**	+++(4)	+++(2)	+++(2)	+++(2)	+++(1)
**Angiogenesis**	vWF^*^	++(4.3 ± 1.1^*^)	++(5.8 ± 1.1)	nd	++(3.9 ± 2.4)	nd
**Intermediate Filament**	**VIM**	+++(2)	++(4)	+++(4)	+++(4)	++++(4)

***TC***= tumor core; ***INV***= invasion; Scored intensity as negative (−), low (+), medium (++), strongly positive (+++) and highly strongly positive (++++) and extent of immunopositivity as 0=negative; 1=1-25%; 2=26-50%; 3=51-75%; 4=>75% positive cells. ^*^ Microvessel Density (mean ± SD). nd = not done.

To validate the role of VIM^+^ cells in PLGG invasion and progression, we collected 5 pairs of matched primary and recurrent PLGGs and compared the expression of GFAP and VIM with IHC (Table 3). In 4 of the 5 tumor pairs (#3319, 9036, 1576, 2022), there were no major differences between primary and recurrent tumors, as nearly all cells were positive for GFAP and VIM (Table 3). In recurrent tumor #1614, cells in the tumor core displayed strong (+++) positivity of GFAP and VIM while a small piece of normal tissue demonstrated significant numbers of VIM^+^/GFAP^−^ tumor cells (Figure 5D, *l* and *o*, *m* and *p*). This identification of VIM^+^/GFAP^−^ cells in the invasive front of this recurrent tumor was in agreement with our observations in the IC-3635PXA tumors.

**Table 3:** Expression of GFAP and Vimentin in paired primary and recurrent pilocytic astrocytomas

Tumor ID	Age /Gender at diagnosis	Time to Recurrent (yr)	GFAP			VIM		
			Primary	Recurrent		Primary	Recurrent	
				TC	INV		TC	INV
1614	18 m/M	15	+++(4^**^)	+++(4)	-	+++(4)	+++(4)	+++(4)
3319	6.5 yr/M	4	+++(4)	+++(4)	^*^	+++(4)	+++(4)	^*^
9036	7.5 yr/M	5.5	+++(4)	+++(4)	^*^	+++(3)	+++(3)	^*^
1576	21 m/M	3	+++(4)	+++(4)	^*^	+++(4)	+++(4)	^*^
2022	2 yr/M	2.5	+++(4)	+++(4)	^*^	+++(4)	+++(4)	^*^

Note: TC= tumor core, INV = invasion, ^*^ no normal tissue; ND= not done. ^**^ Scored intensity as negative (−), low (+), medium (++) and high positive (+++) and extent of immunopositivity as 0=negative; 1=1-25%; 2=26-50%; 3=51-75%; 4=>75% positive cells.

### *In vivo* PDOX progression was paralleled by increased perivascular dissemination

We next examined changes in IC-3635PXA cell invasion in order to guide development of new therapies preventing metastasis. In addition to single cell invasion into neighboring normal brain and neural fibers (Figure 5A, *a* and *b*), there was frequent distant dissemination of VIM^+^ PDOX cells via blood vessels deep into normal brain (Figure 5A, *c* and *d*). Using a straight line reticle (eyepiece micrometer), perivascular migration in mouse cerebral was measured. Migration increased from 140 ± 68.3 μm in passage I, to 182 ± 152, 429 ± 226, and 392 ± 237 μm in passages II, III and IV, respectively (*P* <0.01) (Figure 5B). Longest migratory distance also increased from 350 μm in passage I, to 570, 1,370 and 1,100 μm in passages II, III and IV, respectively. Starting from passage II, we also observed perivascular invasion into brain stem, presumably due to the close proximity of tumor mass (Figure 5B). Since the chances of escaping surgical resection is positively correlated with distance of tumor cell invasion into normal brain, such progressive increases of PXA tumor cell migration into distant normal brains suggested perivascular invasion as a potential cause of tumor recurrence and metastasis.

### Tumor growth triggered intensive reactive gliosis in normal mouse brain

Since human normal brain tissues must be preserved during surgical resection of brain tumors, little is known about the reaction of normal glial cells to the growth of brain tumors, particularly in areas far away from the tumor mass [45]. Using IC-3635PXA, we examined responses of normal glial cells both adjacent to and distant from the primary tumor to infiltration and dissemination of PXA tumor cells. IHC staining for GFAP (which recognizes both human and mouse astrocytes) detected wide-spread presence of GFAP^+^ astrocytes resembling reactive astrocytes [46]. All GFAP^+^ astrocytes cells were negative for human-specific VIM and MT, confirming the murine origin of these cells. Growth of xenograft tumors triggered gliosis in host mouse brains not only at the tumor-brain interface (Figure 5C, *e-i*) but also deep into normal mouse brains beyond the leading edge of tumor invasion, including in the contralateral mouse cerebrum where no human tumor cells were evident (Figure 5C, *f*, *g*, and *i*). Since dynamic interactions between PLGG cells and microenvironment remain poorly defined, PDOX model IC-3635PXA can be used for future understanding of host responses (molecular mechanisms and biological impacts) toward the growth of PXA.

### *In vitro* anti-tumor activities of BRAF V600E inhibitors

**Figure 6:**
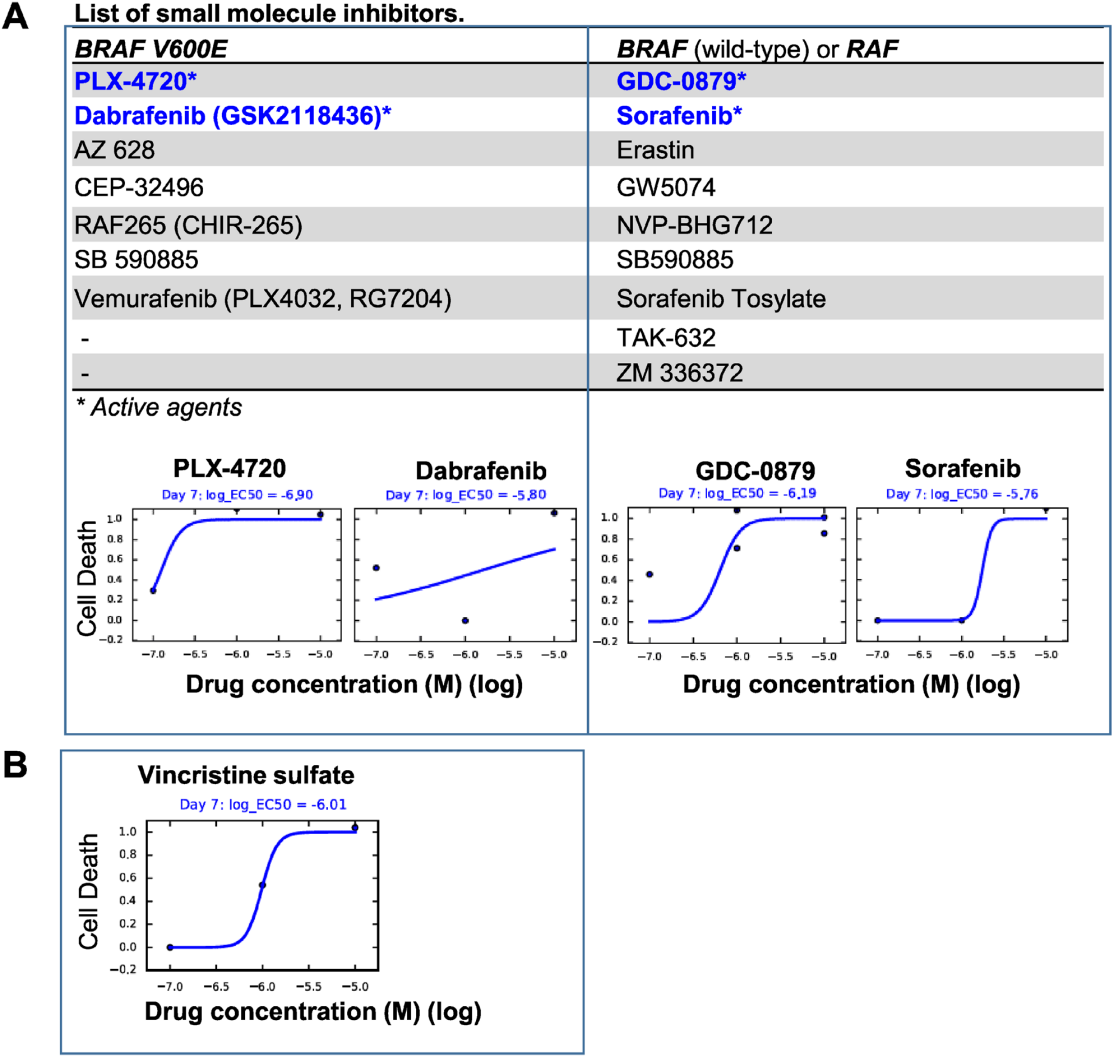
*In vitro* drug testing Cultured 3635PXA cells were exposed to small molecule inhibitors (0.01 to 10 μM) for 7 days and plotted as the fraction of cell killing. **(A)** Two of the 7 Inhibitors targeting *BRAF* V600E (*left panel*) and 2 of the 9 inhibitors against *BRAF* wild-type and *RAF* (*right panel*) were active. Proliferation of the 3635PXA cells were not affected by the remaining agents. **(B)** Chemotherapy agent Vincristine was included as reference.

As a proof of principle, we exposed the cultured BXD-3635PXA cells to vincristine (a chemotherapy agents) and a series of inhibitors that target *BRAF* V600E (n=7), *BRAF* wild-type and/or *RAF* genes (n=9) (Supplementary Table 1) and examined their anti-proliferation activities. After 7 days of *in vitro* treatment (0-10 μM), cell proliferation was suppressed by vincristine (IC50=0.97 μM), 2/7 (28.5%) *BRAF* V600E inhibitors, PLX-4720 (IC50= 0.13μM) and Dabrefenib (IC50=1.58μM), and 2/9 (22.2%) inhibitors that target *BRAF* or *RAF* genes, GDC-0879 (IC50=0.65 μM) and Sorafenib (IC50=1.74 μM) (Figure 6). The remaining 12 inhibitors, including 5 *BRAF* V600E inhibitors, were not active during the treatment period. These data suggested that targeting *BRAF* V600E was effective in suppression BXD-3635PXA cell proliferation. The efficacy of inhibitors, however, was not identical.

## DISCUSSION

In this study, we demonstrated that the tumor take rate of PLGGs is very low as only 1 of the 25 tumors formed transplantable orthotopic xenograft tumors in mouse brains. To determine if the lack of cancer stem cells played a role, we analyzed putative cancer stem cells through FCM analysis of dual-stained putative cancer stem cell markers (CD133 and CD15). While the levels of CD133^+^ cells were much lower (<1%) than a previous report demonstrating 15% CD133^+^ cells in one childhood PA and 8% in two adult grade II ASTs [24], they appear to be correlated with low tumorigenicity of PLGGs. To validate the role of CD133^+^ cells in PLGG tumorigenicity, direct implantation of isolated CD133^+^ PLGG cells should be tested in mouse brains, although low tumor cell abundance coped with low overall tumor cell yields often make it difficult to collect sufficient CD133^+^ cells for this *in vivo* assay. Although 7 of the 22 PLGGs expressed high levels (30% −80%) of CD15^+^ cells, which has been previously detected in medulloblastoma and adult malignant gliomas [16–18], only one of these PLGGs (IC-3635PXA) formed xenografts. Since all of the PLGGs were injected into anatomically-matched locations in mouse brains, our results indicated that the functional role of CD15 in PLGGs might be different from that in the high-grade gliomas. Detailed functional validation is needed for existing and new CSC markers of PLGGs.

While the majority of children with LGGs remain tumor-free, a small fraction of PLGGs do recur over time. Malignant transformation, which is rare in pilocytic astrocytomas (2.4% of 288 patients) [47] and more often in grade II PXA (36%) [48, 49], might have played a role. In this study, we confirmed that IC-3635PXA replicated the histology and key genetic abnormalities of the original patient tumor. More importantly, we showed that, for the first time, the progressive/metastatic growth of a PXA mouse model replicated the progressive and metastatic nature of the originating patient tumor, as evidenced by the increased tumor take rate, elevated cell proliferation and expanded local invasion and perivascular metastasis over serial *in vivo* sub-transplantations. Similar to most recurrent and late stage tumors, no surgery or biopsy was performed in 3635PXA patient tumor at the last recurrence, making it difficult to identify the cellular and molecular changes and/or drivers of tumor recurrence. Using our new PDOX model, we were able to detect the cellular drivers of tumor progression, i.e. GFAP^−^/VIM^+^ tumor cells. Nearly all the xenograft tumor cells became VIM positive starting from passage I with the strongest positivity found in the infiltrating tumor edge. A significant elevation of VIM expression was also confirmed in one paired primary and recurrent PLGGs. Since VIM has been involved in attachment, migration, and cell signaling [50], our results justified additional studies to validate the functional role of GFAP^−^/VIM^+^ tumor cells in PXA (and other PLGGs) recurrence.

Molecularly, we provided new data to support the role of *BRAF V600E* mutation and *CDKN2A* deletion in driving PXA progression. Previous studies have shown that *BRAF V600E* and *CDKN2A* alterations were less commonly observed in PLGG that did not transform [51], but more frequently detected in secondary high grade gliomas. Unlike KIAA1549-BRAF fusion transcripts which were near exclusively expressed in grade I astrocytomas, *BRAF* V600E mutation was detected in 22.6% of grade II-IV tumors but in none of the grade I tumors [36]. In secondary high glade gliomas, *BRAF V600E* and *CDKN2A* deletion were as high as 39% and 57%, respectively [51]. Since 3635PXA patient tumor contained 13% normal diploid cells, the corrected *BRAF* V600E mutation rate increased from 28% to 32.18% (*i.e.,* 28/87=32.18%), it is still much lower than that in the xenograft tumors (>67%). Our long-term follow-up of *in vivo* growth of IC-3635PXA tumors revealed a clear enrichment of *BRAF V600E* mutation frequency and detected a novel a shift of diploid chromosome 9 to trisomy 9 in tumor cells bearing *CDKN2A* deletion. Since copy number of chromosome 9 increased despite *CDKN2A* deletion, addition studies (preferably in more than 1 models) are needed to examine if *CDKN2A* deletion is associated with the *in vivo* progression and *in vitro* growth of neurospheres, and if amplified chromosome 9 is involved in and/or drive tumor progression in PXA. Since *BRAF* V600E mutant gliomas often develop acquired resistance to FDA approved small molecule inhibitor [32], our PDOX model would be a powerful tool to conduct pre-clinical testing of new *BRAF V600E* targeting inhibitors.

Understanding the mode of tumor invasion should provide new clues to guide the development of new therapies preventing metastasis. In addition to migrating as a single cell and along neural fibers, our data showed that PXA xenograft cells achieve distant dissemination through perivascular spaces far beyond the leading edges of intra-parenchymal invasion. The increased frequencies and distances of perivascular migration, which paralleled the xenograft tumor progression over serial *in vivo* sub-transplantations, would render early radiographic detection and surgical resection nearly impossible. However, since these invasive tumor cells in the perivascular space need to breach the blood brain barrier through disruption of astrocytic endfeet, which envelope vessels, to spread away from tumor core, they might have also damaged the integrity of the local blood brain barrier, potentially making themselves vulnerable to chemotherapies. This newly discovered mode of PXA invasion *in vivo* has thus provided a therapeutic opportunity to target the distant perivascular invasion that are left behind after surgery and potentially cause tumor recurrences.

Using this PDOX model, we also had an opportunity to examine the responses of host (normal) brain cells to the hetero-transplanted PXA tumors, particularly in the areas far away from the tumor mass whereas corresponding studies in patients are not feasible. To the best of our knowledge, the widespread reactive gliosis we observed in the normal mouse brain tissues has not been previously described in human PXA or other types of PLGGs. Reactive gliosis, also known as astrogliosis, refers to the morphological and biochemical changes of astrocytes occurring in association with injury or disease. Given the benign nature of PLGGs, it is surprising to detect such an outbreak of hypertrophic and GFAP^+^ reactive astrocytes [45], which have been found to alter the expression or function of neuronal proteins involved in excitability and may serve as drivers of epileptogenesis in acquired epilepsies [52, 53]. Indeed, our 3635PXA patient developed severe epilepsy at tumor recurrence, which highlighted the need and the potential use of our IC-3635PXA model for to decipher the molecular mechanism of reactive gliosis and to identify potential therapeutic targets to improve quality of life by suppressing/preventing epilepsy.

Since development of new therapy is one of the most important goals of model development, we tested the anti-tumor activities of a series of inhibitors specific to *BRAF* V600E or to wild-type *BRAF* or other types of *RAF* gene/pathways *in vitro*. In addition to identify a set of 4 inhibitors (2 each for *BRAF* V600E and *BRAF*) that suppressed cell proliferation, we also found 5 *BRAF* V600E inhibitors and 7 *BRAF* inhibitors that were not active. These findings are interesting as they revealed that differential activities among *BRAF* V600E and wild-type inhibitors, which not only suggested the need of “personalized” drug testing, but also highlighted the potential use of our model for the examination of underlying mechanisms of action and resistance.

In conclusion, our studies demonstrated that PLGGs has very low tumorigenic capacity in the brains of SCID mice, which is similar to the frequency of tumor progression and recurrence in pediatric patients. Low abundance of CD133^+^ cells appears to be correlated with such low tumor take rate. The PDOX model IC-3635PXA not only replicated the histological and molecular phenotypes, but also, and more interestingly, evolved *in vivo* replicating the progressive growth of the originating patient tumor. Using this novel PDOX model, we have identified VIM^+^GFAP^−^ cells as candidate cellular drivers, *BRAF V600E* mutation and *CDKN2A* deletion (in cells with trisomy and quadrisomy chromosome 9) as key molecular changes that mediated the progression, invasion and migration of the xenograft tumors over long term *in vivo* subtransplantations, and showed that a subset of *BRAF* V600E inhibitors were indeed active in suppression cell proliferation *in vitro*. This novel model will serve as an important resource to support further biological and pre-clinical studies (such as *BRAF V600E* mutation) in pediatric PXA.

## MATERIALS AND METHODS

### Childhood LGG tumor tissues

Freshly resected LGG tumor specimens from 36 children undergoing craniotomy at Texas Children's Hospital and member hospitals of the Texas and Oklahoma Pediatric Neuro-Oncology Consortium were obtained for this study (Table 1). Signed consent was obtained prior to sample acquisition following Institutional Review Board-approved protocols. As described previously [7, 8], fresh tumor tissues were washed and dissociated with the Automatic Tissue Dissociator (Miltenyi Biotec), followed by collagenase/halogenase enzymatic digestion.

Tumor #3635 was obtained from a 9 year-old girl receiving subtotal resection of an extensive left temporal tumor. Pathologically, it was diagnosed as PXA with ganglioglioma component (WHO grade II), *BRAF V600E* mutation and low cell proliferation index (<2%). Two month later, she received more complete subtotal resection. The tumor, however, progressed along with disseminated neuraxis metastasis 5 months from the initial diagnosis. Despite treatment with palliative craniospinal radiatioin along with daily adjuvant temozolomide chemotherapy during radiation therapy, she developed severe epilepsy, post-chemoradiotherapy pancytopenia, speptic shock and passed away 7 months after diagnosis.

### Flow cytometry

PLGG tumor cells were labeled with APC-conjugated human CD133 antibody and FITC-conjugated human CD15 antibody (Miltenyi Biotec), or isotype control antibodies at 4°C for 15 minutes in FCM buffer comprised of DPBS, 0.5% BSA and 2 mM EDTA. After washing, cells were re-suspended in FCM buffer containing 2 μg/mL propidium iodide (PI) and analyzed with a LSR II flow cytometer and Kaluza Analysis Software Version 1.3 (Beckman Coulter). Dead cells were excluded by PI staining.

### *In vitro* growth of PLGG cells

For neurosphere assays, dissociated PLGG tumor cells were plated at clonal density (1,500 cells/100 μL) and incubated in serum-free media consisting of Neurobasal media, N-2 and B-27 supplements (0.5x each) (Life Technologies), human recombinant basic fibroblast growth factor (bFGF), epidermal growth factor (EGF) (50 ng/mL each) (R&D Systems) [8, 54], and 200 units/mL penicillin/streptomycin. Additional cells were seeded in DMEM media supplemented with 10% fetal bovine serum (FBS) and 200 units/mL penicillin/streptomycin. Cells were incubated in 5% CO_2_ at 37°C. Media were changed every three days and cell growth examined under phase contrast microscopy.

### Direct orthotopic transplantation of patient tumor cells into mouse brain

NOD/SCID mice were bred and housed in a pathogen-free animal facility. All experiments were conducted following an Institutional Animal Care and Use Committee-approved protocol. Mice of both gender, aged 6-8 weeks, were anesthetized with sodium pentobarbital (50 mg/kg, i.p. injections). Tumor cells from 25 PLGGs were re-suspended in DMEM growth medium at 5 × 10^7^ live cells/mL and injected (1 × 10^5^ cells in 2 μL) orthotopically into mouse brains as described previously [7, 8, 55]. Cerebellar tumors were implanted into right cerebellum (1 mm to the right of the midline, 1 mm posterior to the lamboidal suture, and 3 mm deep), and cerebral PLGGs into right cerebral hemisphere (1 mm to the right of the midline, 1.5 mm anterior to the lamboidal suture, and 3 mm deep) via a 10 μL26-gauge Hamilton Gastight 1701 syringe needle. Animals were monitored daily for signs of neurological deficits. Mice without neurological deficits after 12 months were euthanized and examined for tumor formation.

### Hematoxylin and eosin (H&E) and Immunohistochemical (IHC) Staining

Whole mouse brains were harvested, fixed in zinc formalin, paraffin-embedded, and serially sectioned. H&E staining was performed on every 20 sections. IHC staining was performed using Vectastain Elite ABC kit (Vector Laboratories) or Mouse on Mouse Elite Peroxidase Kit (Vector Laboratories) as described previously [7, 8, 55]. Primary antibodies included human-specific monoclonal antibodies against mitochondria (MT) (1:150) (EMD Millipore Corporation), Vimentin (VIM) (1:200) (Dako North America,), glial fibrillary acidic protein (GFAP) (1:100) (Abcam), and rabbit anti-von Willebrand Factor (vWF) (1:500) (EMD Millipore Corporation).

### Pyrosequencing

*BRAF* V600E mutation status was examined through pyrosequencing. Following genomic DNA extraction a PCR reaction was set-up (40 cycles, 58°C annealing temperature) using primers Forward 5′-GGCCAAAAATTTAATCAGTGGAA-3′ and Reverse 5-Bio-CTTCATAATGCTTG CTCTGATAGG-3′. PCR reaction amplified a 236-bp genomic fragment spanning *BRAF* codon 600 on exon 15. Pyrosequencing was performed on a PSQ 96 (Qiagen) as previously described [56]. The pyrogram sequence was analyzed using sequencing primer PySeq 5′-CCACTCCATC GAGATT-3′ and dispensation order –CTAGCATGCTGT–. Output was recorded as antisense 5′→3′ sequence and % A calculated using pyromark analysis software. Incorporation of non-wild type nucleotide at position 7 with allelic frequency >10 was considered positive for mutation.

### Florescence *in situ* hybridization (FISH)

To determine *CDKN2A* deletion, Vysis LSI CDKN2A SpectrumOrange/CEP 9 SpectrumGreen Probes were applied to paraffin sections from patient and xenograft tumors and cultured xenograft cells. Paraffin sections were de-paraffinized and cultured cells cytospun onto positively charged slides and treated with protease. Slides and probe mixture were co-denatured at 75°C for 5 minutes and placed in the hybrite machine at 37°C overnight. After post-hybridization wash, slides were counterstained with DAPI and examined under florescence microscopy. Data analysis was performed by counting cells using CEP 9 probe labeled with SpectrumGreen, which hybridizes to alpha satellite sequence on chromosome 9.

### *In vitro* drug treatment

Cultured BXD-3635PXA cells in serum-free media were seeded 50 μL per well into 384-well plate using Multidrop dispenser (Thermo Fisher Scientific) and incubated at 37°C for 24 hr before the investigational compounds (50 nL and 5 nL from 10 mM stock, and 50 nL from 0.1 mL stock) were transferred using Echo550, an acoustic liquid handler from Labcyte (San Jose, CA). The plates were then incubated at 37°C in a CO_2_ incubator for 7 days. To estimate cell proliferation, 5 μL of CCK8 was added to each well, incubated for 4 hrs before the absorbance was measured at 450 nm using 650 nm as references.

### Statistical analysis

Differences between two groups were analyzed with student *t* test. *P* values <0.05 were considered significant.

## SUPPLEMENTARY MATERIALS TABLE




