# Supplementary material for: Xenotransplantation of pediatric low grade gliomas confirms the enrichment of BRAF V600E mutation and preservation of CDKN2A deletion in a novel orthotopic xenograft mouse model of progressive pleomorphic xanthoastrocytoma
Source: Oncotarget. 2017 Sep 8;8(50):87455–71. doi: 10.18632/oncotarget.20713 (PMC5675646; doi:10.18632/oncotarget.20713)
Supplement: Supplementary file 1 [file oncotarget-08-87455-s001.pdf]

Xenotransplantation of pediatric low grade gliomas confirms the enrichment of *BRAF* V600E mutation and preservation of *CDKN2A* deletion in a novel orthotopic xenograft mouse model of progressive pleomorphic xanthoastrocytoma

SUPPLEMENTARY MATERIALS

Supplementary Table 1: List of small molecule inhibitors of *BRAF* V600E, *BRAF* (wild-type) and *RAF*

See supplementary File 1
