# Supplementary material for: Xenotransplantation of pediatric low grade gliomas confirms the enrichment of BRAF V600E mutation and preservation of CDKN2A deletion in a novel orthotopic xenograft mouse model of progressive pleomorphic xanthoastrocytoma
Source: Oncotarget. 2017 Sep 8;8(50):87455–71. doi: 10.18632/oncotarget.20713 (PMC5675646; doi:10.18632/oncotarget.20713)
Supplement: Supplementary file 2 [file oncotarget-08-87455-s002.docx]

| **Supplemental Table 1**. List of small molecule inhibitors of *BRAF* V600E, *BRAF* (wild-type) and *RAF*. | | | |
| --- | --- | --- | --- |
| **No.** | **Agent** | **Description** | **EC50 (µM)** |
| ***BRAF V600E Inhibitor*** | | |  |
| 1 | PLX-4720 | A potent and selective inhibitor of *BRAF* V600E with IC50 of 13 nM in a cell-free assay, equally potent to c-Raf-1(Y340D and Y341D mutations), 10-fold selectivity for *BRAF* V600E than wild-type *BRAF*. | 0.13 |
| 2 | Dabrafenib (GSK2118436) | A mutant *BRAF* V600E specific inhibitor with IC50 of 0.8 nM in cell-free assays, with 4- and 6-fold less potency against *BRAF* (wild-type) and c-*Raf*, respectively. | 1.58 |
| 3 | AZ 628 | A new pan-*Raf* inhibitor for *BRAF*, *BRAF* V600E, and c-Raf-1 with IC50 of 105 nM, 34 nM and 29 nM in cell-free assays, also inhibits VEGFR2, DDR2, Lyn, Flt1, FMS, etc. | n/a* |
| 4 | CEP-32496 | A highly potent inhibitor of *BRAF* (V600E/wild-type) and c-Raf with Kd of 14 nM/36 nM and 39 nM, also potent to Abl-1, c-Kit, Ret, PDGFRβ and VEGFR2, respectively; insignificant affinity for MEK-1, MEK-2, ERK-1 and ERK-2. Phase 1/2. | n/a |
| 5 | RAF265 (CHIR-265) | A potent selective inhibitor of c-Raf/*BRAF*/*BRAF* V600E with IC50 of 3-60 nM, and exhibits potent inhibition on VEGFR2 phosphorylation with EC50 of 30 nM in cell-free assays. Phase 2. | n/a |
| 6 | SB 590885 | Potent *BRAF* inhibitor (Kd = 0.3 nM). Selective for *BRAF* against 46 other kinases (Ki app values are 0.16 and 1.72 nM for *BRAF* and c-Raf respectively). Decreases anchorage-independent growth of melanoma cell lines. Inhibits ERK phosphorylation and proliferation in tumor cells expressing *BRAF* V600E. | n/a |
| 7 | Vemurafenib (PLX4032, RG7204) | A novel and potent inhibitor of *BRAF* V600E with IC50 of 31 nM in cell-free assay. 10-fold selective for *BRAF* V600E over wild-type *BRAF* in enzymatic assays and the cellular selectivity can exceed 100-fold. | n/a |
| ***BRAF and RAF inhibitors*** | | |  |
| 8 | GDC-0879 | A novel, potent, and selective *BRAF* inhibitor with IC50 of 0.13 nM in A375 and Colo205 cells with activity against c-Raf as well; no inhibition known to other protein kinases. | 0.65 |
| 9 | Sorafenib | A multikinase inhibitor of Raf-1, *BRAF* and VEGFR-2 with IC50 of 6 nM, 22 nM and 90 nM in cell-free assays, respectively. | 1.74 |
| 10 | Erastin | Selective for cells expressing oncogenic RAS. Also Ferroptosis activator; induces oxidative, non-apoptotic cell death in tumors by modulating mitochondrial voltage-dependent anion channels (VDAC). | n/a |
| 11 | GW5074 | A potent and selective c-Raf inhibitor with IC50 of 9 nM, no effect on the activities of JNK1/2/3, MEK1, MKK6/7, CDK1/2, c-Src, p38 MAP, VEGFR2 or c-Fms is noted. | n/a |
| 12 | NVP-BHG712 | A a specific EphB4 inhibitor with ED50 of 25 nM that discriminates between VEGFR and EphB4 inhibition; also shows activity against c-Raf, c-Src and c-Abl with IC50 of 0.395 μM, 1.266 μM and 1.667 μM, respectively. | n/a |
| 13 | SB590885 | A potent *BRAF* inhibitor with Ki of 0.16 nM in a cell-free assay, 11-fold greater selectivity for *BRAF* over c-Raf. | n/a |
| 14 | Sorafenib Tosylate | A multikinase inhibitor of Raf-1, *BRAF* and VEGFR-2 with IC50 of 6 nM, 22 nM and 90 nM in cell-free assays, respectively. | n/a |
| 15 | TAK-632 | A potent pan-Raf inhibitor with IC50 of 8.3 nM and 1.4 nM for *BRAF* (wild-type) and C-Raf in cell-free assays, respectively, showing less or no inhibition against other tested kinases. | n/a |
| 16 | ZM 336372 | A potent and selective c-Raf inhibitor with IC50 of 70 nM, 10-fold selectivity over B-RAF, no inhibition to PKA/B/C, AMPK, p70S6, etc. | n/a |
| Note: n/a= not active. | | |  |
